# Moral Preferences

**DOI:** 10.1007/s12115-016-0027-3

**Published:** 2016-04-01

**Authors:** Lisa Bortolotti, Anneli Jefferson

**Affiliations:** Philosophy Department, University of Birmingham, ERI Building, B15 2TT Edgbaston, UK

**Keywords:** Moral psychology, Preference satisfaction, Duty, Hedonism, Egoism, Values, Self-image, Positive illusions, Happiness

## Abstract

In this brief response to Etzioni’s paper we argue that satisfying one’s preferences and seeking to live up to one’s moral standards are not incompatible ways of living one’s life, and that choosing to act morally need not involve self-sacrifice.

Etzioni’s interesting article starts with the contrast between *satisfiers*, who only care for their own happiness and act to maximise their pleasure and minimise their pain, and *affirmers*, who seek to live in accordance with their moral values. In the rest of the article, Etzioni argues that human nature is characterised by a constant wrestling between satisfying one’s desire for happiness and honouring one’s moral commitments. As what people want to do is heavily conditioned by the society they live in, Etzioni suggests that, for the common good, people should be encouraged, or better, socially conditioned, to pursue affirmation over satisfaction. This is because human agents are naturally motivated to pursue happiness but find it harder to be motivated to honour their moral commitments.

We find the initial contrast between *selfish* happiness-seeking agents and *selfless* virtue-seeking agents unconvincing. The distinction between the quest for happiness and the quest for morality drawn by Etzioni is a fairly modern and contested one. For example, Aristotle’s *eudaimonia* can famously be translated both as happiness and as flourishing. Clearly, there are historical precedents for seeing the morally good life and the happy life as one and the same.

What agents want, ultimately, is to satisfy their preferences. Some of such preferences are about states of affairs that bring agents pleasure. It is not a surprise that most human agents care about gaining recognition, enjoy a good meal, take pride in their own achievements and in those of their partners, offsprings, and friends. Other preferences are about states of affairs that do not necessarily bring agents pleasure in the short term. Typically, human agents strive to be the type of person they would like themselves to be, and want to make the world around them closer to the world they would like to live in. Some of what it is for agents to fulfil their moral commitments can be identified with the satisfaction of preferences in this latter category: agents may want to be more altruistic and compassionate, or they may want their society to be fairer and more inclusive, and the measures they take to fufil such goals may not bring immediate pleasure.

But this does not rule out that some of an agent’s moral commitments can be furthered by satisfying preferences in the former category: just because agents take pleasure from the satisfaction of some of their preferences, this does not mean that satisfying those preferences is deprived of moral significance or does not further their moral aims. Take the pleasure that a mother gets from her son’s good academic performance. Her preference for her son to do well may have been an important factor in motivating her to act in a number of selfless and helpful ways, such as supporting her son at difficult times, and putting his interests before her own. Whether or not she gets pleasure from his achievements, the fact that she wanted him to achieve and helped him to do so is morally significant.

One way of characterising the aspect of human nature Etzioni focuses on in his article is to acknowledge that typical agents are individuals who have a variety of preferences. But Etzioni cautions against describing both the pursuit of pleasurable experiences and the fulfilment of moral commitments as preference satisfaction, for several reasons. Etzioni is concerned that, if we talk about preference satisfaction in both cases, the motivation to fulfil moral commitments may be reduced to a self-serving one, also aimed at maximising pleasure, but in a less direct way (e.g., the son who diligently takes care of his aging father does so not out of genuine compassion and concern for his father, but because by doing so he feel better about himself).

The worry is that the reduction would be wildly implausible, because fulfilling moral commitments involves considerable self-sacrifice. This matters to Etzioni’s final recommendations: his view is that, given that affirmation typically involves pain, it “tends to decay if left to its own devices”, whereas the satisfaction of hedonistic preferences preserves its strength through time. Thus, Etzioni argues in the end that society should intervene to support affirmation. In what follows, we suggest that the psychological mechanisms responsible for affirmation and for the pursuit of hedonistic preferences are znot obviously distinct, and that the motivation for affirmation can be effectively sustained by the desire to preserve and enhance a positive concept of oneself as a unified, coherent, and causally efficacious agent.

## Is it Implausible to Characterise Affirmation as a Form of Satisfaction?

Etzioni's worry is that if we see morality as just another kind of desire satisfaction we take away what is distinctly moral about morality. This is a recurring topic. If we talk about affirmation in terms of preference satisfaction, aren’t we reducing moral ambitions to an ultimately pleasure-seeking and self-serving strategy? The thought Etzioni articulates here is that all altruistic behaviour is basically selfish. If doing good to others gives agents pleasure and ignoring the need of others is painful to agents, then agents only act morally  because it makes them happy.

One of the most elegant refutations of the claim that “if doing what is good makes one happy and acting immorally makes one unhappy, then all moral actions are selfish” has been provided by David Hume. He argues that, in order for one’s doing good to others to have a positive effect on one’s well-being, one first needs to value the wellbeing of others. That is, the selfish pleasure one gets from doing good to others is contingent on one’s altruistic motivations.

There are mental passions, by which we are impelled immediately to seek particular objects, such as fame, or power, or vengeance, without any regard to interest; and when these objects are attained, a pleasing enjoyment ensues, as the consequence of our indulged affections. (…) Were there no appetite of any kind antecedent to self-love, that propensity could scarcely ever exert itself, because we should, in that case, have felt few and slender pains or pleasures and have little misery or happiness to avoid or pursue. (Hume 1998, EPM, Appendix 2, SBN 301).

It follows that we can concede that even if one fulfils moral goals as part of satisfying one’s preferences, this does not make altruistic preferences selfish in any meaningful sense. Rather, agents need to care about doing the right thing to derive some satisfaction from it. The selfish motivation piggybacks on the altruistic motivation.

Importantly, this account does not only apply to moral behaviour. As Hume points out, many things are pleasurable to agents because agents value them, and would not be pleasurable to them if they did not value them. He illustrates this using the example of the desire for fame. “Nature must, by the internal frame and constitution of the mind, give an original propensity to fame, ere we can reap any pleasure from that acquisition.” (Hume 1998, EPM, SBN 301) This shared feature of moral and non-moral preferences undermines the claim that there is a special problem with talking about moral goals as preferences.

It is not obvious that the psychological mechanisms behind satisfying hedonistic preferences are distinct from those that motivate people to satisfy their moral preferences. For example, there is evidence that performing altruistic acts, such as giving to charity, recruits the same brain areas associated with rewarding experiences, such as receiving a financial reward. (cf. Moll et al. 2006) The hypothesis that agents experience pleasure when they perform a moral action is consistent with the point Etzioni himself makes, when he discusses the empirical studies showing that performing altruistic acts such as volunteering is perceived as rewarding. Furthermore, as outlined in our initial example, there are many cases where hedonistic and moral preferences are not in competition. This brings us to Etzioni’s central claim that acting morally involves pain.

## Does Affirmation Typically Involve Pain?

Etzioni appears to be suspicious of people who enjoy doing good things. He says that "activities that serve higher needs may be prosocial but nevertheless tend to be amoral". We take it that he has two reasons for making this claim. One is the selfishness objection discussed and dismissed above.^1^ The other is his claim that acting morally typically involves pain. Is it true that pursuing happiness involves maximising pleasure whereas fulfilling moral commitments involves sacrifice? We will argue that a) living up to one’s moral standards is not always painful, and that b) there are non-moral self-realisation goals which also require painful sacrifices. Consequently, moral goals and preferences do not differ from non-moral ones in a principled way.

One example Etzioni offers for moral action that requires sacrifice is the case where someone, call her Jill, would like to go see a movie but ought to visit a friend in hospital. In this case, there is indeed a sacrifice to be made. But assume that the Jill’s desire to visit her ill friend is stronger than her desire to see a movie. In that case, Jill’s inclination and her duty coincide. Absent a further argument, it would seem petty to claim that the her act is any less moral just because she also enjoys it. Taken together with the empirical evidence that people find doing what they perceive to be morally right rewarding, this example shows that painfulness may be a feature of moral conduct, but it does not need to be.

Furthermore, one may also make sacrifices to satisfy a non-moral preference. Think of the following scenario: Tim ought to go to the gym, but he would like to stay on the couch and eat chocolate. The ‘ought’ in the sentence above is not a moral ought, it is an ought that is contingent on Tim’s goals such as staying healthy, controlling his weight, and so on. Just as in the case of the moral sacrifice, Tim may not enjoy the gym much, but still gets a warm glow of satisfaction after completing his goal. Again, the contrast between satisfying moral and non-moral goals is much less pronounced than Etzioni makes it out to be.

But maybe the difference is best understood as one of degree. After all, Etzioni claims that living up to one’s moral commitments is *typically* painful, not that it is *always* painful. While there is room to argue that realising one’s moral goals requires no bigger sacrifice than realising ambitious self-actualization goals (such as writing a novel or climbing mount Everest), it is indubitably the case that living up to one’s moral standards frequently does require one to sacrifice some other interest. What is more, it is the costliness of moral action that frequently leads one to fall short of one’s moral ideals. There is a significant body of research, both in the literature on positive illusions and in that on situationism, which shows that people’s moral self-image is unduly positive (Brown 2012; Sedikides, Meek, Alicke, and Taylor 2014). People overestimate the power of their good intentions and underestimate the force of situational factors that make doing the right thing costly, either because doing the right thing is inconvenient or for some other reason (Dunning 2004; Epley and Dunning 2000; Tenbrunsel, Diekmann, Wade-Benzoni, and Bazerman 2010).

## Supporting Moral Preferences and Conduct

Is it plausible that the motivation to pursue hedonistic preferences sustains itself whereas the motivation to pursue moral preferences is destined to fade if not incentivised by the state or society? Etzioni claims that affirmation is integral to human nature and section 5 of his paper, which reviews evidence of systematic affirming behaviour, shows that affirming behaviour is fairly robust. Moreover, one's  desire to preserve a positive and largely coherent concept of oneself may be instrumental to avoiding acting in ways that would violate one's moral commitments. However, specific moral norms may be culture-relative or even depend on the individual’s set of values.

We take both Etzioni’s article and our discussion to show that the desire to act morally is part of the normal human psychological make-up. Because moral conduct does frequently require sacrifices, agents may fall short of their moral ideals. More worryingly, due to positive illusions and the tendency to see themselves in an unrealistically positive light, people may not even notice that their behaviour does not match their moral standards, or they may be unrealistically optimistic about the likelihood of them doing what is right. It is in helping agents to live up to their moral standards that the state or society has a role to play in incentivizing moral conduct, and in disincentivizing immoral and criminal behaviour. The significance of the intervention of the state or society can be seen in Etzioni’s example of people who were interviewed about their propensity to commit illegal acts. They refrained from committing crimes both for moral reasons and in order to avoid negative repercussions. Social sanctioning is indeed a powerful source of motivation.

## Footnotes

1 One might wonder what the problem would be with altruism being selfishly motivated, even if the claim were true. Presumably, Etzioni worries that moral behaviour would then become contingent on our inclinations and there would be no reason for the person who happens not to care about the wellbeing of others to behave morally.
